# Supplementary material for: African cratonic lithosphere carved by mantle plumes
Source: Nat Commun. 2020 Jan 3;11:92. doi: 10.1038/s41467-019-13871-2 (PMC6941964; doi:10.1038/s41467-019-13871-2)
Supplement: Supplementary file 1 — Supplementary material [file 41467_2019_13871_MOESM1_ESM.pdf]

Supplementary information for African cratonic lithosphere carved by mantle plumes

Celli et al.

Supplementary Note 1

We observe that kimberlites and lamproites in Africa do not tend to be located close to the boundaries of the cratonic mantle lithosphere, as proposed previously[2, 3]. We compared the location of the kimberlite and lamproite samples with those of the cratonic boundaries, mapped the average S-wave velocity anomalies in the 110-150 km depth range and, alternatively, using Vs gradients (Supplementary Fig. 5). We defined the boundary areas as all points within a 200-km wide band just outside the craton boundary (defined using different, alternative shear-velocity anomaly thresholds) (Supplementary Fig. 5a). In order to test the robustness of the patterns, we repeated the comparison with a different definition of the boundary area (+/- 150 km from the boundary, 300 km width in total) (Supplementary Fig. 5b). These wide boundary regions, with alternative different widths and differently defined boundary lines, account for differences in possible definitions of cratonic boundaries and also for the finite resolution of our tomography. With all tested margins, no preferential distribution of the samples within the boundary regions is observed, indicating that cratonic margins do not appear to control their distribution (Supplementary Fig. 5a-f). Comparing the kimberlite and lamproite distributions to the gradients of the S-wave velocity anomalies, we can also observe that most of the samples occur where the gradients are low (Supplementary Fig. 5g,h,i), lower than continental average. This confirms that the African kimberlites and lamproites are not distributed preferentially at or near the craton-lithosphere boundaries.

Supplementary Note 2

To verify that the lithospheric fragmentation we observe is not caused by artifacts due to insufficient or uneven data coverage, we conducted a series of resolution tests, including spike tests (Figs. S7, S8) and a targeted structure test (Supplementary Fig. 9). In the structure test, the synthetic input model simulated the broad anomalies beneath the major cratons seen in earlier tomographic models. A velocity anomaly of 250 m/s was assigned to all model nodes within the broad-craton boundaries[4] in the 7-250 km depth interval. A velocity anomaly of -200 m/s was assigned to the nodes beneath the Ethiopian Rift Valley in the 7-250 km depth range, in order to simulate the East African Rift Zone. The results (Supplementary Fig. 9) show accurate recovery of both the shape and amplitude of the anomalies, with minor differences in the simulated Kalahari craton, whose northern part in the test was very narrow. The tests show that if the cratonic lithospheres were the broad, monolithic features seen in earlier, smoother models, based on smaller datasets, then our tomography would have retrieved them as such.

Supplementary Note 3

Because some kimberlite pipes yield numerous samples, the locations of these pipes may get excessive weight in the distributions we examine. We have thus re-sampled the kimberlite and lamproite data on grids and tested a range of different grid cell sizes, including 0.03, 0.1 and 0.5 degree cell sizes (Supplementary Fig. 10). As the cell size increases beyond 0.03 degrees, not only samples from the same pipes but also those from different, closely-spaced pipes get averaged together, given the width of a pipe typically a few hundred meters[1]. This leads to a bias, with excessive weight for isolated samples. The patterns we observe, however, are robust and present both without the resampling and with resampling with various cellsizes: most diamondiferous kimberlites and lamproites in Africa are not in areas with the high shear velocity anomalies in the lithospheric depth range characteristic of thick cratonic lithosphere.

Supplementary Figures

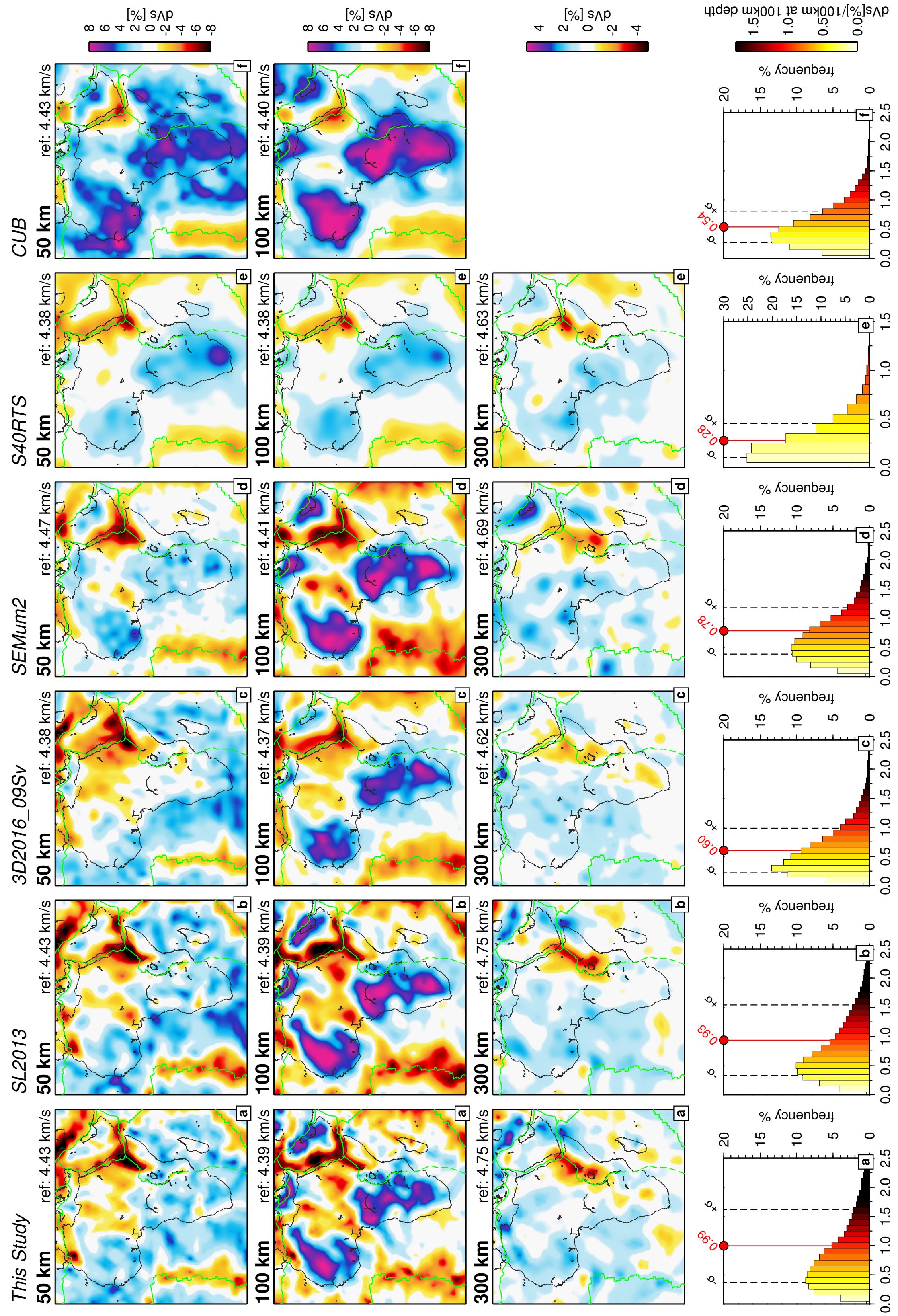

Supplementary Figure 1: Caption next page.

Supplementary Figure 1: (Previous Page) Comparison of our tomographic model AF2019 with other tomographic models. Tomographic mapviews at 50, 100 and 300 km depth are shown for the following models: a) AF2019; b) SL2013[5], c) 3D2016_09Sv[6]; d) SEMum2[7]; e) S40RTS[8]; f) CUB[9]. All models are plotted as S-wave velocity anomalies with respect to the mean velocity at depth, shown on the top right corner of each model plot. Major plate boundaries are plotted as green lines (solid lines: verified; dotted lines: proposed). The bottom row shows histograms of the S-wave velocity anomaly gradients in continental Africa; averages are plotted in red, standard deviations as dashed black lines.

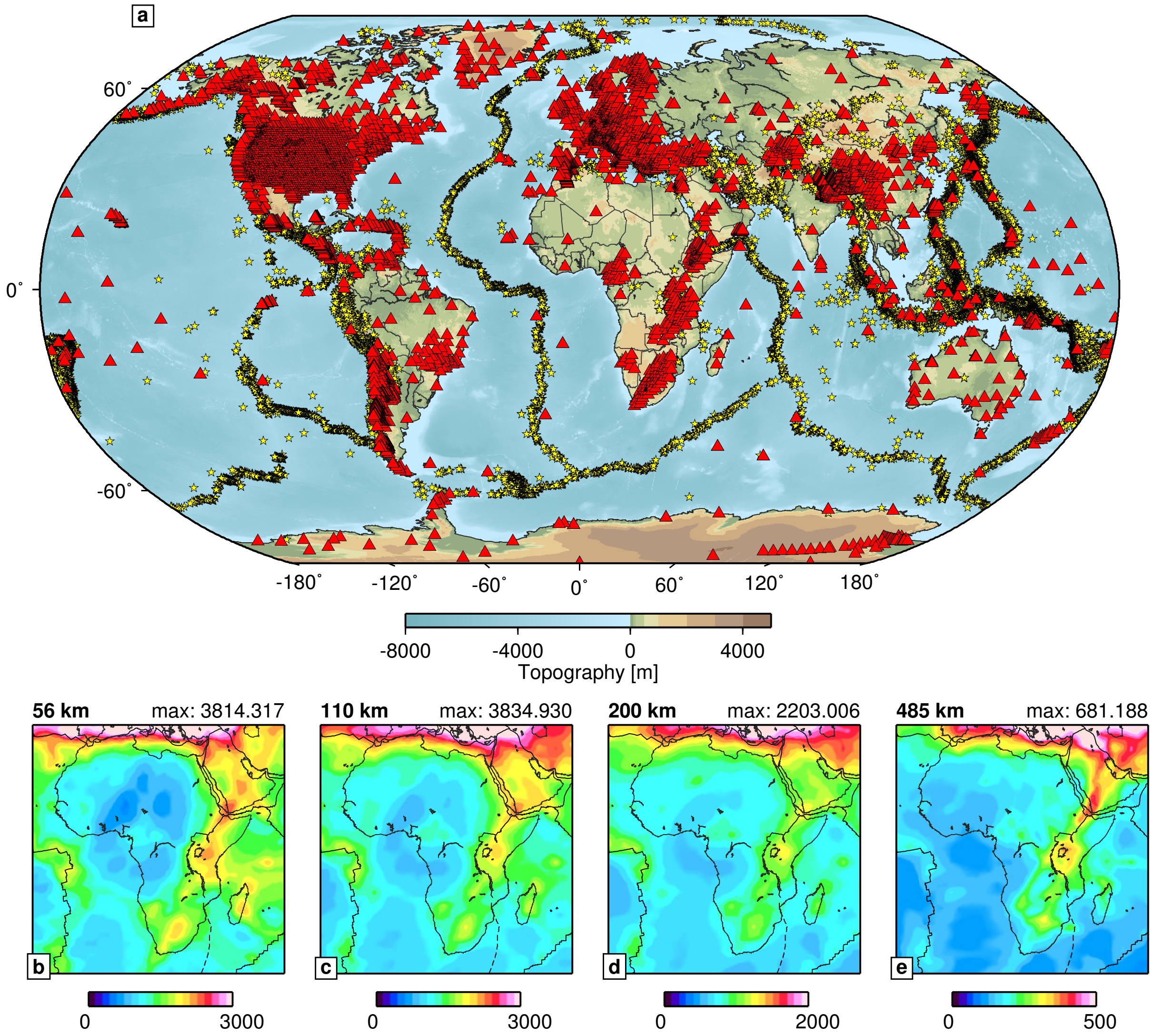

Supplementary Figure 2: The data sampling we used to compute our tomographic model. a): Global distribution of seismic stations (red triangles) and earthquakes epicentres (yellow stars) used in our waveform tomography. b-e): The sums of the columns of the sensitivity matrix at four selected grid node depths (56, 110, 200, 485) that represent most of the depth range investigated. The column sums yield an adimensional measure of the relative sampling of the structure at each node by our waveform data set.

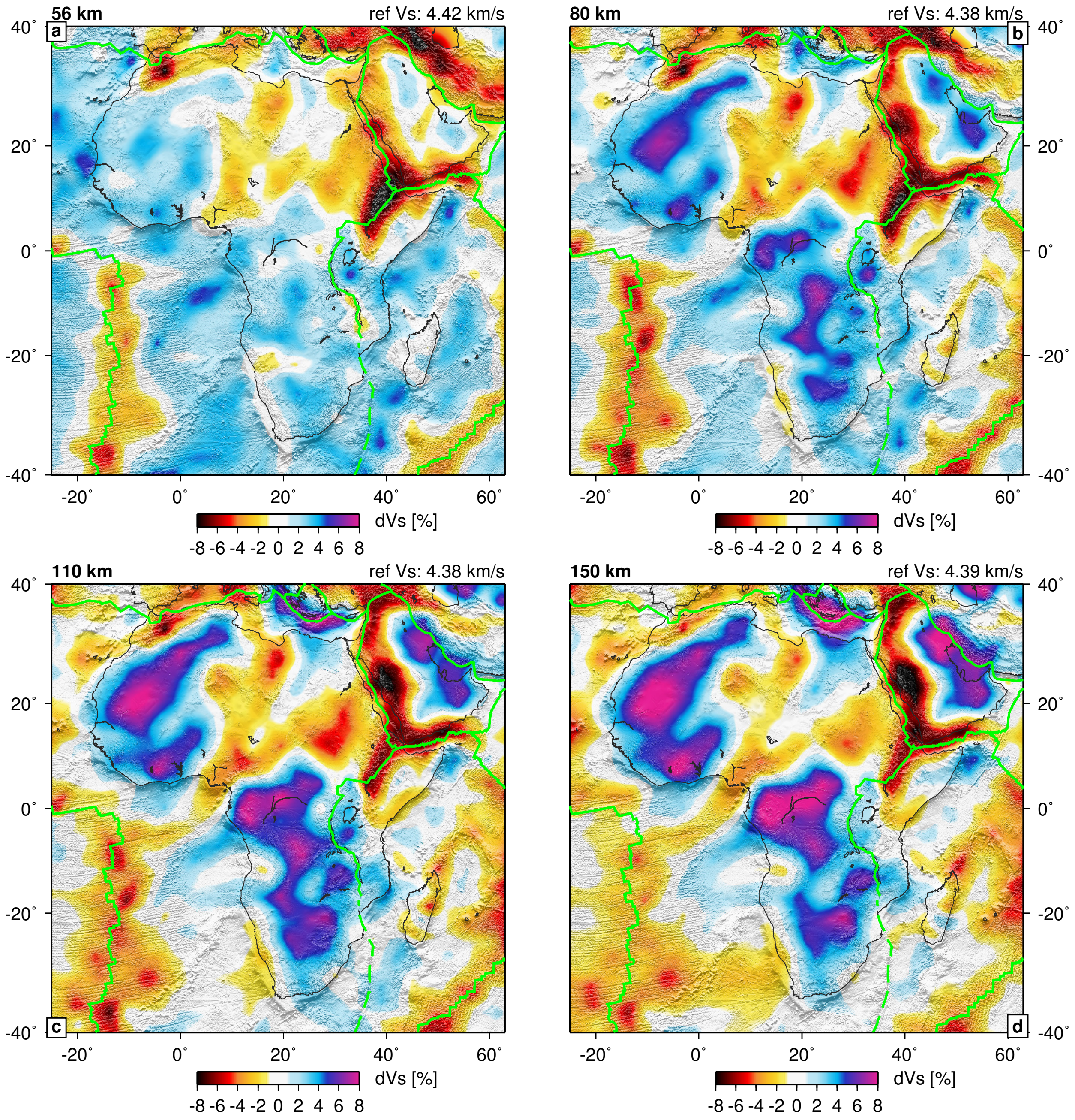

Supplementary Figure 3: S-wave velocity anomalies at four depths in the shallow upper mantle: a) 56 km; b) 80 km; c) 110 km; d) 150 km. The depth is indicated above each panel on the left, with the reference velocity on the right. Topography is superimposed as shading.

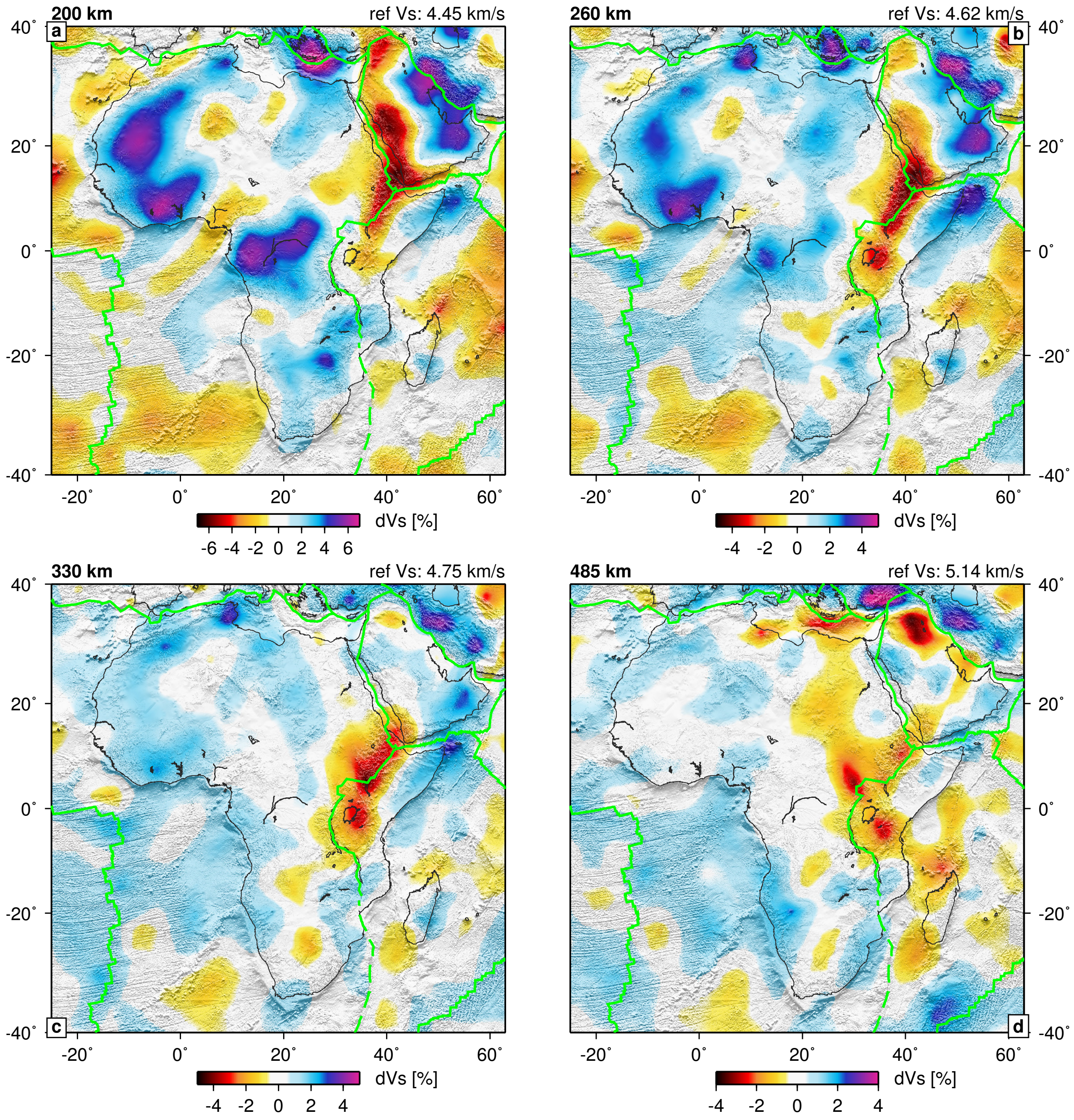

Supplementary Figure 4: S-wave velocity anomalies at four depths in the deep upper mantle and transition zone: a) 200 km; b) 260 km; c) 330 km; d) 485 km. The depth is indicated above each panel on the left, with the reference velocity on the right. Topography is superimposed as shading.

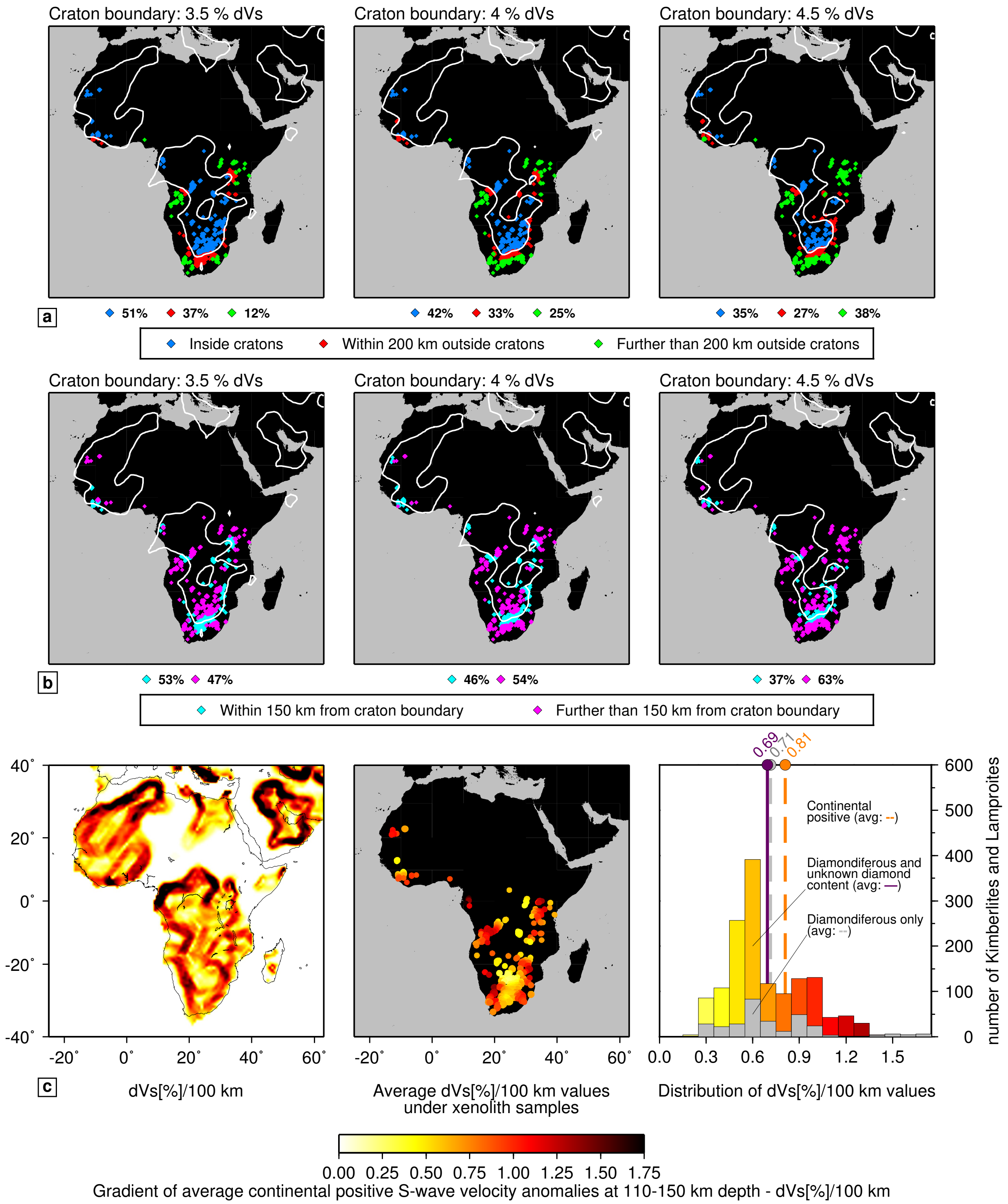

Supplementary Figure 5: Caption next page.

Supplementary Figure 5: (Previous page) Kimberlites and lamproites in Africa do not tend to be located close to the boundaries of the cratonic mantle lithosphere. This is shown by the kimberlite and lamproite locations relative to cratonic boundaries, defined either using S-wave velocity anomaly contours (a, b), or the gradient of the average S-wave velocity anomaly in the lithosphere (c). In panel a), samples are grouped as: inside craton boundaries (blue); within 200-km wide area just outside the craton boundary (green); further than 200 km outside the craton boundary (red). In panel b), samples are grouped as: within +/-150 km from a craton boundary (300-km wide area around the boundary) (cyan) and further than 150 km from a craton boundary (magenta). For each contour, percentages of each population are indicated at the bottom of the plot. Panels c) show the gradient of the S-wave velocity anomaly averaged over the 110-150 km depth range (for clarity, only areas with positive dVs are included), its values under the kimberlite and lamproite samples, and the histogram of these values for diamondiferous and diamondiferous plus "unknown samples". The average gradient values for all positive-anomaly areas, for diamondiferous plus unknown samples, and for diamondiferous samples only are indicated in orange, purple and grey, respectively.

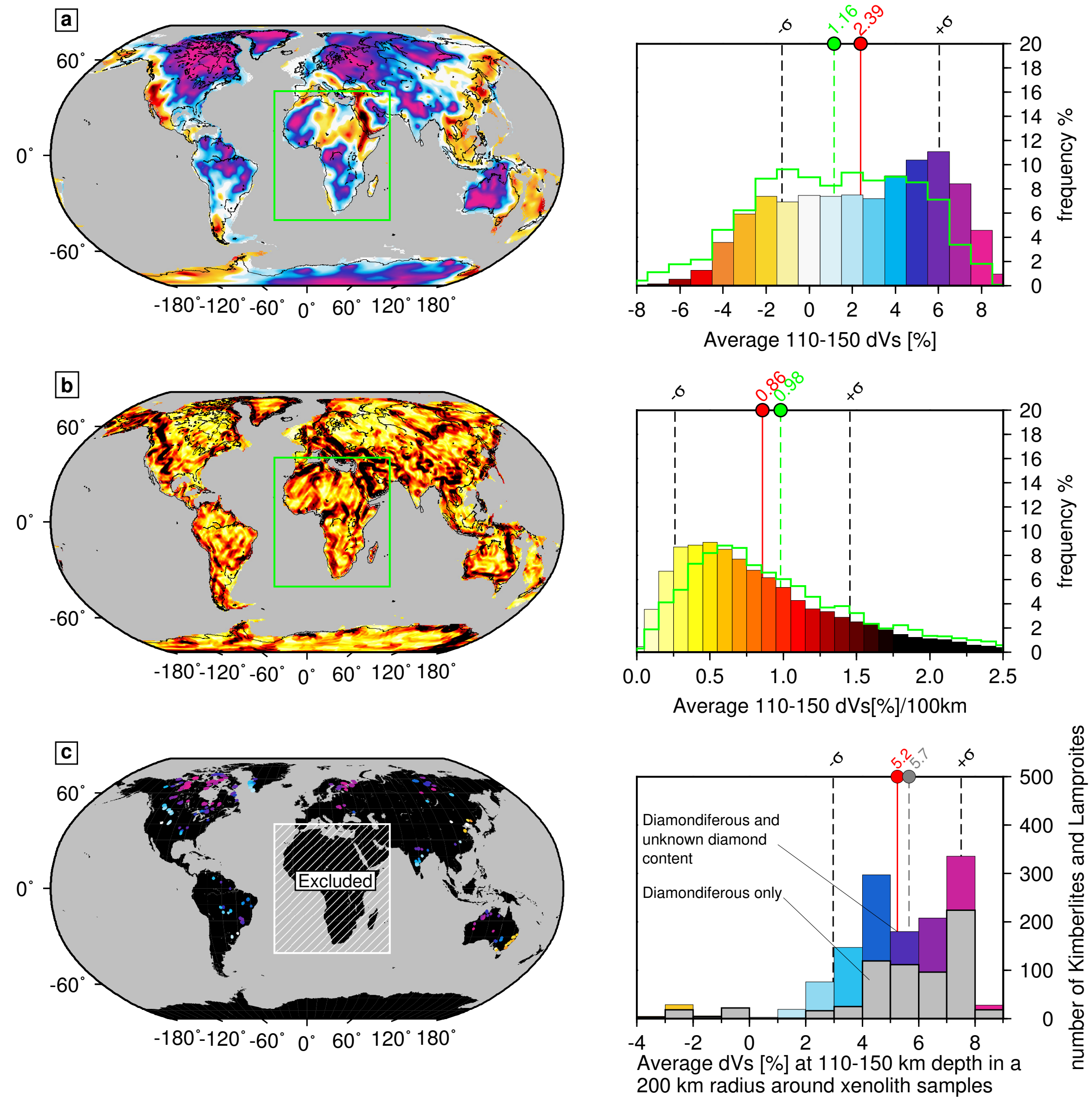

Supplementary Figure 6: Continental lithosphere and kimberlites outside Africa. dVs average at 110-150 km depth (a), its gradient for the continental regions (b) and its average value in a radius of 200 km around non-African kimberlite samples only (c). In the histograms a) and b), coloured bins represent the global values, whereas the green bins are for Africa only. Average values for the whole globe and Africa are shown in red and green, respectively. Standard deviations for all continents are shown as black dashed lines. The extent of the "Africa" area is shown as green boundaries on the map views a), b) and as a white striped area in map c). In the histogram c), coloured bins are for kimberlite and lamproites that can be either diamondiferous or of unknown diamond content, whereas the grey bins are for diamondiferous samples only.

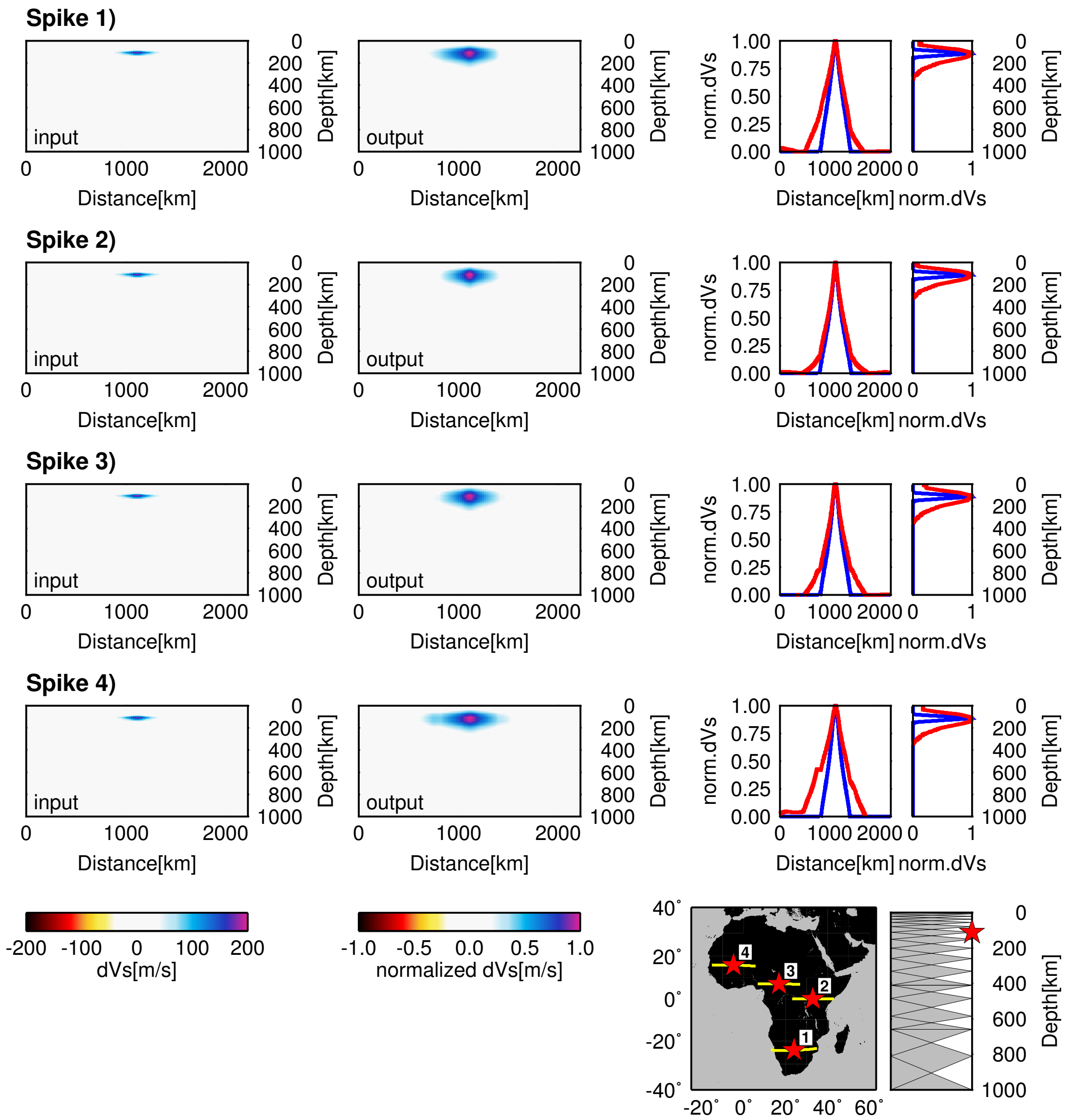

Supplementary Figure 7: Synthetic spike test at 110 km. For each of the four spikes, we plot, left to right, the input synthetic model, the model resulting from the inversion and a horizontal and vertical velocity profiles through both of them (blue: input, red: output). All spikes are constructed by assigning a 200 m/s S-wave velocity anomaly to a single node of the model. The locations of the spikes on the map and on the depth axis are shown with red stars. On the map, the locations of the cross-sections are plotted in yellow. Vertical basis functions of the model are plotted in the depth plot.

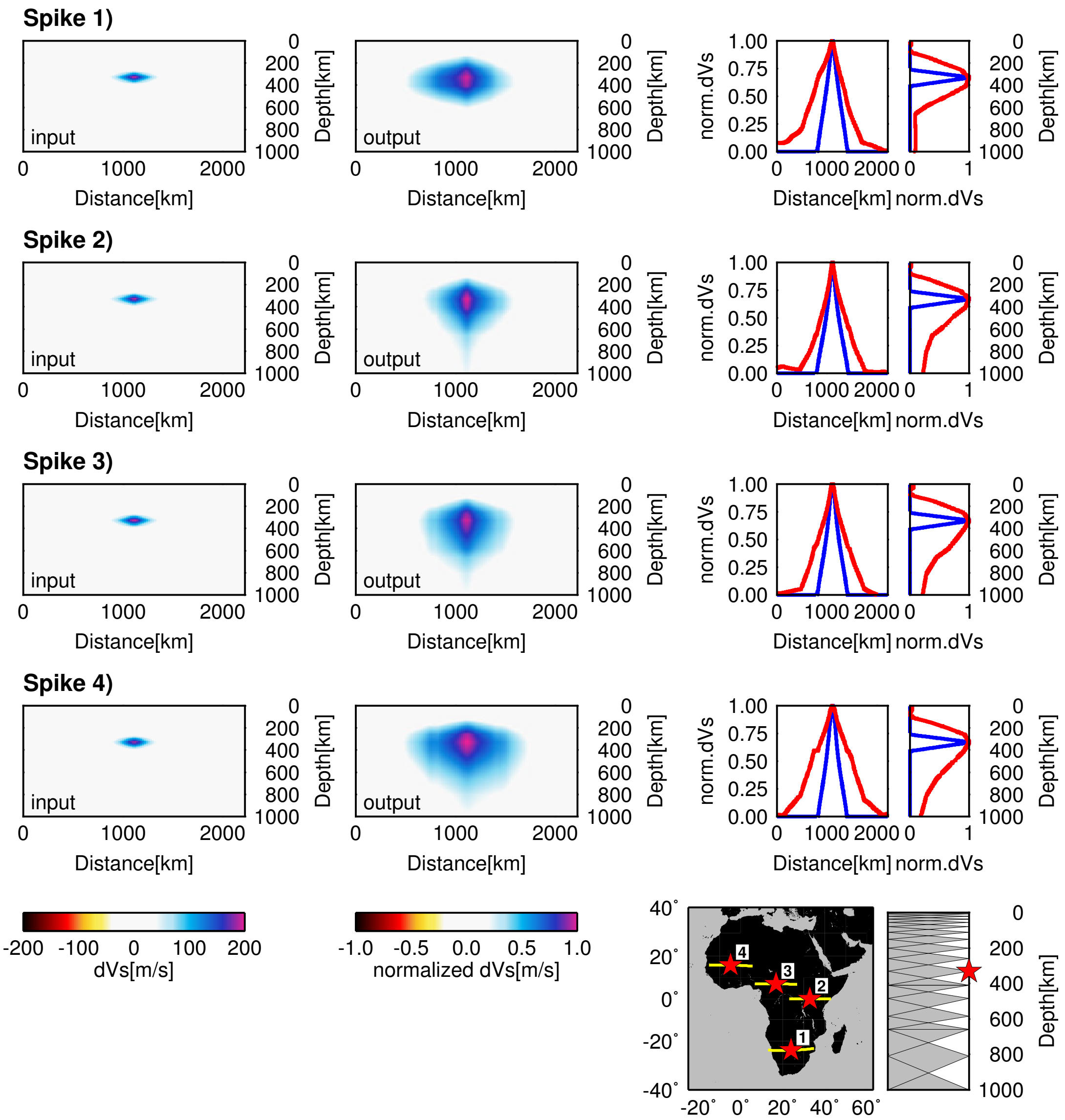

Supplementary Figure 8: Synthetic spike test at 330 km. For each of the four spikes, we plot, left to right, the input synthetic model, the model resulting from the inversion and a horizontal and vertical velocity profiles through both of them (blue: input, red: output). All spikes are constructed by assigning a 200 m/s S-wave velocity anomaly to a single node of the model. The locations of the spikes on the map and on the depth axis are shown with red stars. On the map, the locations of the cross-sections are plotted in yellow. Vertical basis functions of the model are plotted in the depth plot.

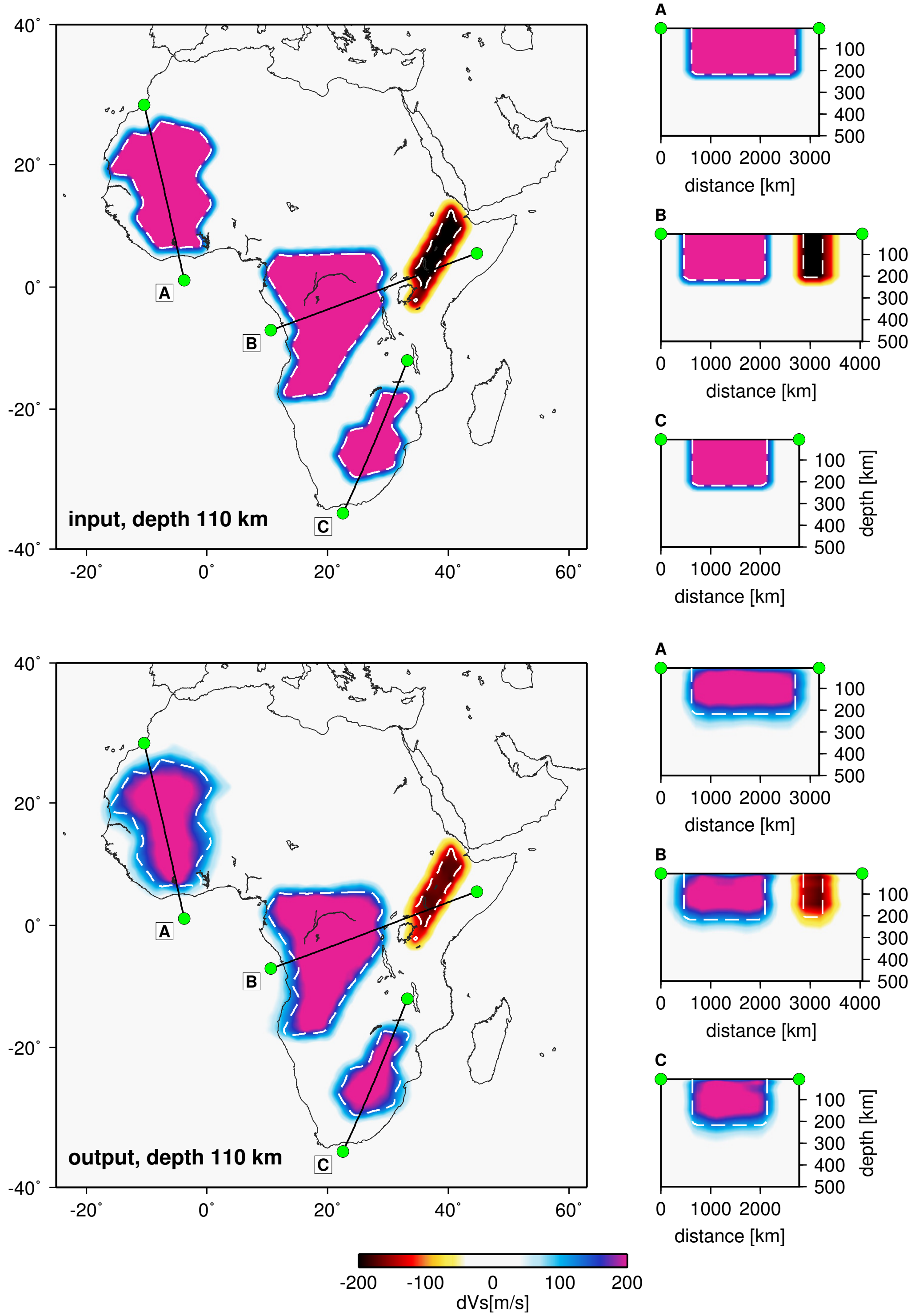

Supplementary Figure 9: Synthetic structure test simulating broad anomalies beneath the major cratons and the Ethiopian Rift Valley as seen in previous tomographic models. Top model shows the input synthetic structure, bottom model shows the output of the inversion. Both models are shown both as 110 km depth horizontal slice (left panels) and three vertical cross-sections cutting through the synthetic structures (right panels). Location of the cross-sections is plotted on the horizontal slices. Contours of the synthetic input are plotted as dashed white lines.

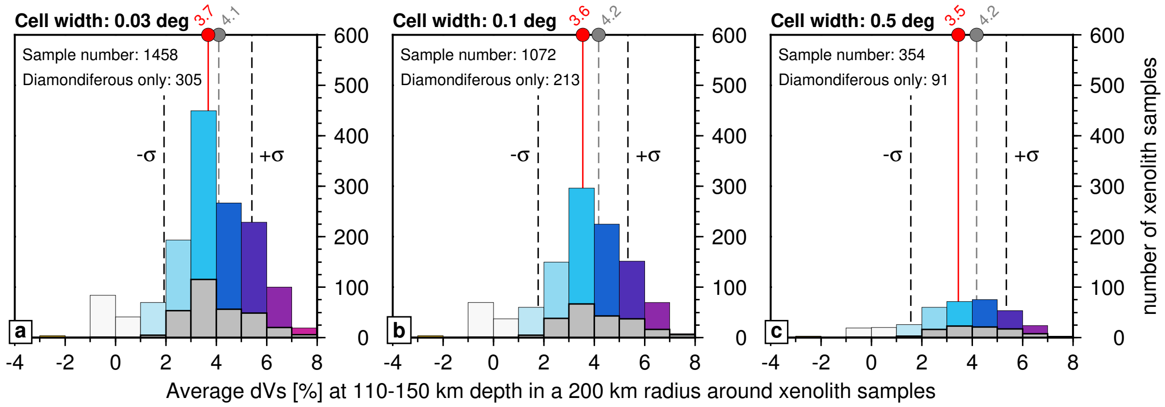

Supplementary Figure 10: The effect of resampling the kimberlite and lamproite data on a grid, so as to avoid a sampling bias in the histograms due to numerous samples coming from some of the kimberlite pipes. Results of the resampling with the grid cell sizes of 0.03, 0.1 and 0.5 degrees are shown in the left, middle and right panels, respectively. The histograms are computed using average S-wave velocity anomaly over the 110-150 km depth range, also averaged across a circle of a 200 km radius around each sample. The combined diamondiferous and unknown-content samples are shown as coloured bars, with their average plotted as red line and circle. Diamondiferous samples only are plotted as grey bars, with their average plotted as grey line and circle. The number of samples remaining after the regridding is specified in the top left corner of each panel.

Supplementary References

[1] Kjarsgaard, B. a. Kimberlite Pipe Models : Significance for Exploration. *Ore Deposits and Exploration Technology* 667–677 (2007).

[2] McKenzie, D. & Priestley, K. The influence of lithospheric thickness variations on continental evolution. *Lithos* **102**, 1–11 (2007).

[3] Faure, S., Godey, S., Fallara, F. & Trépanier, S. Seismic architecture of the archean north american mantle and its relationship to diamondiferous kimberlite fields. *Economic Geology* **106**, 223–240 (2011).

[4] Begg, G. C. *et al.* The lithospheric architecture of Africa: Seismic tomography, mantle petrology, and tectonic evolution. *Geosphere* **5**, 23–50 (2009).

[5] Schaeffer, A. J. & Lebedev, S. Global shear speed structure of the upper mantle and transition zone. *Geophysical Journal International* **194**, 417–449 (2013).

[6] Debayle, E., Dubuffet, F. & Durand, S. An automatically updated S -wave model of the upper mantle and the depth extent of azimuthal anisotropy. *Geophysical Research Letters* **43**, 674–682 (2016).

[7] French, S. W., Lekic, V. & Romanowicz, B. Waveform tomography reveals channeled flow at the base of the oceanic asthenosphere. *Science* **342**, 227–30 (2013).

[8] Ritsema, J., Deuss, A., Van Heijst, H. J. & Woodhouse, J. H. S40RTS: A degree-40 shear-velocity model for the mantle from new Rayleigh wave dispersion, teleseismic traveltime and normal-mode splitting function measurements. *Geophysical Journal International* **184**, 1223–1236 (2011).

[9] Shapiro, N. M. & Ritzwoller, M. H. Monte-Carlo inversion for a global shear velocity model of the crust and upper mantle. *Geophysical Journal International* **151**, 81–105 (2002).
